# A Revisit to the Formation and Mitigation of 3-Chloropropane-1,2-Diol in Palm Oil Production

**DOI:** 10.3390/foods9121769

**Published:** 2020-11-29

**Authors:** Farrah Aida Arris, Vincent Tiang Soon Thai, Wan Nabilah Manan, Mohd Shaiful Sajab

**Affiliations:** 1 Research Center for Sustainable Process Technology (CESPRO), Faculty of Engineering and Built Environment, Universiti Kebangsaan Malaysia, Bangi, Selangor 43600, Malaysia; farrahaidaarris@gmail.com (F.A.A.); a159833@siswa.ukm.edu.my (V.T.S.T.); p95338@siswa.ukm.edu.my (W.N.M.); 2 Department of Chemical and Process Engineering, Faculty of Engineering and Built Environment, Universiti Kebangsaan Malaysia, Bangi, Selangor 43600, Malaysia

**Keywords:** 3-chloropropane-1,2-diol (3-MCPD) measurement, 3-MCPD mitigation, palm oil, refined bleached deodorized (RBD) palm oil

## Abstract

Process-based contaminants in food—particularly in vegetable oils—have been a topic of interest due to their potential health risk on humans. Oral consumption above the tolerable daily intake might result in health risks. Therefore, it is critical to correctly address the food contaminant issues with a proper mitigation plan, in order to reduce and subsequently remove the occurrence of the contaminant. 3-monochloropropane-1,3-diol (3-MCPD), an organic chemical compound, is one of the heat- and process-induced food contaminants, belonging to a group called chloropropanols. This review paper discusses the occurrence of the 3-MCPD food contaminant in different types of vegetable oils, possible 3-MCPD formation routes, and also methods of reduction or removal of 3-MCPD in its free and bound esterified forms in vegetable oils, mostly in palm oil due to its highest 3-MCPD content.

## 1. Introduction

3-monochloropropane-1,2-diol or 3-MCPD is one of the food processing contaminants included in the group of chloropropanols. 3-MCPD is a glycerol chlorohydrin, which is formed when one out of three hydroxyl group (-OH) in glycerol, is substituted by one (1) chlorine (-Cl) atom [1]. 3-MCPD is developed once fat- and salt-based foods are processed at high temperature such as vegetable oils, breads, shortenings and soy sauce. The chloropropanol exists in different forms, either as free substance, esters of fatty acids or both [2]. 3-MCPD can also be formed from a reaction between hydrochloric acid (HCl) and residual vegetable fat during acid-hydrolyzed vegetable protein (HVP) [3]. 3-MCPD has a molecular formula of C_3_H_7_ClO_2_, a molar mass of 110.539 g mol^−1^, a density of 1.32 g cm^−3^, and a boiling point of 213 °C [4]. It exists either in free form, or in bound form called 3-MCPD esters [5,6]. Up to now, many researchers have identified the occurrence of 3-MCPD in different foodstuffs, where most of them are fat-containing foodstuffs or food that has passed through cooking processes using cooking oil, or refining process [7,8,9,10,11,12,13,14,15,16]. 3-MCPD exists in a monoesterified form that consists of about 15% of the total amount of 3-MCPD [17]. Additionally, the 3-MCPD mono to diesters ratio has suggested a marginal contribution of the 3-MCPD esters exposure. The other three isomers, 2-MCPD (2-monochloropropane-1,3-diol), 1,3-DCP (1,3-dichloro-2-propanol) and 2,3-DCP (2,3-dichloro-1-propanol) are commonly found at considerably lower concentrations, or none at all [18,19]. Both free and esterified forms of 3-MCPD were found in HVP, which is a flavoring agent and is commonly used in soy sauce production.

The main issue with 3-MCPD relies on the toxicological data on its carcinogenicity after testing on rats, with results showing infertility in rats, destruction of the immune system and small vacuolated lesions in the brainstem area of the rats [20,21,22,23,24,25,26,27,28]. Genotoxicity, which can be described as a chemical agent that destroys genetic information within a cell and subsequently leads to mutation. This mutation activity may ultimately lead to cancer generation [29]. Findings show that 3-MCPD is a genotoxic agent in vitro and a non-genotoxic agent in vivo [30,31]. Following the raising concern with regards to 3-MCPD potential health risk to human, in 2001, the Joint Food and Agricultural Organization (FAO)/World Health Organization (WHO) Expert Committee on Food Additives or in short JEFCA, and the European Commission (EC) Scientific Committee on Food decided to set a provisional maximum tolerable daily intake (PTDI) of 2 µg 3-MCPD per kg body weight per day [32]. Commission Regulation (EC) in 2006 has set the regulatory limit of 2 µg 3-MCPD per kg bodyweight, with a maximum level of 20 µg per kg in HVP and soy sauce [33]. In 2011, International Agency for Research on Cancer (IARC) categorized 3-MCPD as a group 2B, which translates 3-MCPD as a compound possibly carcinogenic to human [34]. Back in 2017, the Scientific Opinion of the EFSA Panel on Contaminants in the Food Chain (CONTAM Panel) has set the tolerable daily intake (TDI) of 0.8 µg per kg body weight (bw) for 3-MCPD and its fatty acid esters, as compared to 4.0 µg per kg bodyweight specified by JEFCA in 2016 [35]. The food safety issue arises from 3-MCPD is expected to bring unavoidable implications to major vegetable oil producers, especially palm oil for instance Malaysia and Indonesia. Moreover, 85% of the palm oil produced globally are used for food applications [36]. This paper will review the occurrence, formation and mitigation of 3-MCPD in vegetable oils especially in palm oil due to its highest 3-MCPD content.

## 2. 3-MCPD in Vegetable Oils

### 2.1. General Extraction Process

Vegetable oils are edible oils made from fats extracted from plants, specifically from its seeds or fruits. Edible oils are mainly composed of triacylglycerols (TAGs), accompanied by lower levels of diacylglycerols (DAGs), monoacylglycerols (MAGs) and free fatty acids (FFA). Edible oils also contain minor components such as phosphatides, sterols, fatty alcohols, fat soluble vitamins and other compounds [37]. Classical examples of vegetable oils produced from its seeds or beans are soybean oils and canola or rapeseed oils, whereas an example of vegetable oil produced from its fruits is palm oil, which is also one of the most widely used vegetable oil in the world. Other types of vegetable oils include cottonseed oil, corn oil, olive oil, coconut oil, peanut oil, safflower oil and sunflower oil. Palm oil and soybean oil cover more than 50% of the total world production for vegetable oil, with 61.40 and 49.04 million metric tons in the year 2014 and 2015, respectively [38]. Classification of vegetable oils can be made based on several methods. For instance, classification by composition characterization according to the amount of fatty acids [39], classification using the Fourier transform infrared (FTIR) method [39,40], classification according to the concentration of saturated fatty acids, which can be performed using the laser-induced breakdown spectroscopy (LIBS) method [41], and classification based on oil fractions performed using high-resolution carbon-13 nuclear magnetic resonance (^13^C NMR) spectroscopy [42]. The classification of vegetable oils can also be performed according to its sterol profiles as well as using gas chromatography method [43,44].

Oils made from seed should be stored in good conditions to avoid alterations caused by insects or molds on damaged seeds, as well as to prevent or delay lipid oxidation, which is known to be influenced by enzymes, temperature, humidity and oxygen. The tricks and techniques that reduce the seeds’ humidity in dryers and that store them under controlled atmospheres have been applied to avoid such deterioration. The cleaning of oil seeds is useful to remove impurities, to protect the downstream processing equipment and also to regulate the bulk density, being the preparation process based on volumetric quantities [45].

Oil extraction is the unit operation performed to separate oil from the solid portion of the seed. The oil seeds are processed by one of the following three different types of processes; screw press extraction, prepress solvent extraction and solvent extraction [46]. Oils obtained from oil seed processing have to be refined before consumption. The objective of refining is to remove unwanted compounds such as phosphatides, FFA, pigments, odor, flavors, metals, pesticides, and aromatic hydrocarbons, preserving the desirable components of the crude oils such as vitamins without significant losses of the major glyceride components [46].

The principal steps of the vegetable oil refining process include degumming, neutralization, bleaching, and deodorization. Degumming is designed to eliminate phosphatide and mucilaginous materials whereas neutralization is a process aimed to remove FFA. Bleaching serves a purpose to eliminate pigments such as chlorophylls and carotenoids as well as molecules that promotes oxidation such as hydroperoxide, and deodorization is a process to remove the off-flavor volatile substances. Another refining process called dewaxing is targeted to eliminate the higher melting acylglycerols and long chain aliphatic hydrocarbons [47].

**Figure 1:**
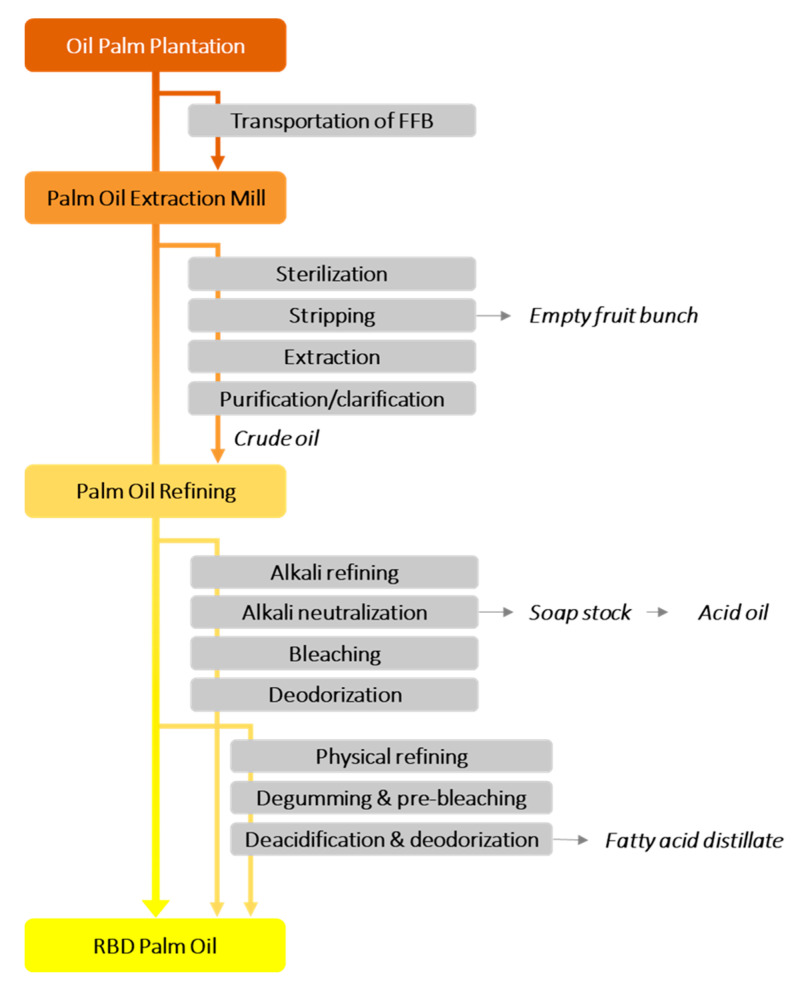
An example of full processing flow chart for a palm oil refining process from fresh fruit bunch (FFB) to the refined bleached deodorized (RBD) palm oil.

The oil refining processes can be classified as chemical and physical, which mainly differs by the technology used for FFA removal. For physical refining, FFAs are removed by distillation process during deodorization, which in this case, phosphatides and other impurities must be eliminated before steam distillation [48]. In chemical refining, most of the impurities in the FFA are removed with an alkaline solution during neutralization, usually using sodium hydroxide (NaOH) [49]. An overall summary of the palm oil refining process is an example of vegetable oil processing and depicted in Figure 1 [50,51,52,53].

It is important to remove impurities from the refined vegetable to ensure that the quality requirements are met and the oils are safe for consumption. The quality of the extracted oils determines the grade of the premium oil, with low FFA level indicates a high quality of refined oil, which can be used for oral consumption for instance as cooking oil [54,55]. Therefore, proper processing and cleaning steps are essential in the effort to decrease the amount of undesired compounds that can adversely impact the overall experience of customer acceptance in terms of palate sensitivity, appearance, food safety, and a prolonged shelf life.

### 2.2. Occurrence and Formation

Back in 1983, Gardner research group was one of the first researchers to identify 3-MCPD occurrence in cooking oils and to report that 3-MCPD esters occurs in rapeseed oils mixed with aniline and refined with HCl [56]. Since then, many researchers have worked on identifying 3-MCPD, mostly in the form of fatty esters, in various vegetable oils such as rapeseed oil, olive oil, soybean oil, sunflower oil, safflower oil and palm oil [9,17,53,54,55,56,57,58,59,60,61,62,63]. For vegetable oils, the amount of 3-MCPD in free forms ranging from less than 0.003 mg per kg; whereas, 3-MCPD in bound form was reported to have less than 0.1 mg per kg. The lowest concentration of bounded form of 3-MCPD was observed in virgin oils, with less than 0.1 mg per kg [62].

For refined olive oil, an esterified form of 3-MCPD amounts up to 1.5 mg per kg [64], whereas in fat mixture containing palm olein, an estimated amount of 2.5 mg per kg of 3-MCPD were found [17,48]. Refined fruit oils—such as palm oils and olive oils—generate higher levels of 3-MCPD esters compared to seed oils such as rapeseed oil or maize oil [62]. Rapeseed oil was reported to produce a lesser amount of 3-MCPD esters compared to palm oil, potentially due to the lower amount of the alleged precursors such as chlorine and diglycerides. In addition, refined oils show a significantly higher fatty acid ester content as opposed to non-refined oils, which are mostly undetected or below tolerable daily limit. A heat pretreatment of the seeds or fruits, for instance a roasting process, can contribute to a heightened amount of 3-MCPD contaminant in non-refined oils [48,62]. In addition, 3-MCPD ester is also reported to be undetectable in virgin and unrefined oils, and has the tendency to be produced during high thermal refining process such as deodorization [58]. The formation of free 3-MCPD depends hugely on temperature, lipid content, glycerol, salt-containing compound, and water [65]. TAGs with its fats and oils components comprise of FFA, MAGs and DAGs. The degree of the 3-MCPD formation relies heavily on the concentration of the precursor raw material prior to processing. Rapeseed oils, sunflower oil, olive oil and soybean oil contain between 1% to 3% DAGs, whereas in palm oils, the amount of DAGs ranging between 6% and 10%. Crude coconut oil, palm oil and palm kernel oils were reported to contain up to 7% amount of FFAs [65]. It has also been reported that palm oil and palm-derived oils, for example palm stearin and palm olein, contain the highest 3-MCPD ester content compared to other vegetable oil [57].

The refining process of palm oil can be categorized into two types, namely physical and chemical refining. Briefly, the physical refining involves the three main steps of degumming, bleaching and deodorization. While chemical refining, on the other hand, requires an additional refining step called neutralization. Neutralization is a chemical refining process, which converts the FFA in the degummed oil to soapstock, a mixture of fatty acid soaps, salts, phospholipids, impurities and neutral oil [66]. NaOH, potassium hydroxide (KOH), sodium bicarbonate (NaHCO_3_) and sodium carbonate (Na_2_CO_3_) are the main alkaline reagents used in this process. The process of neutralization involves gravity settling followed by separation of soap stock from neutralized oil. Finally, the residues of alkaline are washed off from the oil system using hot water [65].

During the initial stage of the refining process, the crude palm oil (CPO) undergoes degumming process, which includes treatment of CPO with water, salts, enzymes, caustic soda, or dilute acids for the removal of phosphatides, waxes, pro-oxidants, and other impurities into gums, which are insoluble in oil. Next, the insoluble gums can be separated from the system through filtration, centrifugation or gravitational settling. There are several different types of degumming methods, namely water degumming, acid degumming, dry degumming and enzymatic degumming [66]. Water degumming involves removing phosphatides in the CPO using water. Acid degumming consists of CPO treatment with phosphoric acid or citric acid (with 2–5% water) at temperature range around 80 to 95 °C. In dry degumming, CPO is mixed with 0.05% to 0.10% concentrated phosphoric acid and heated to temperature around 80 to 110 °C. In enzymatic degumming, on the other hand, a phospholipid degrading enzyme called phospholipase is used.

Next, the degummed oil is sent for bleaching treatment with bleaching earth. During the bleaching process, the oil is heated with various types of bleaching clay or bleaching earth at a high temperature of between 85 to 110 °C under vacuum condition (720 mmHg to 760 mmHg). The process helps to remove metal traces, color pigments, phosphatides and other impurities [66]. As a result, the compounds such as phospholipids, colorants, soaps, and other contaminants are removed from the oil system to yield the desired characteristics of refined vegetable oils. Some examples of bleaching clay or bleaching earth that are typically used are acid-activated bleaching earth, fuller’s earth, and activated charcoal [67].

Palm oil is highlighted as the major contribution in the global vegetable oils market share. Palm oil has a wide potential application in the fields of food, cosmetic, pharmaceutical, chemical and biofuel [68,69,70]. As one of the most efficient oil crops economically, oil palm exhibits a relatively low cultivation cost, an ability to tolerate climate change and a high crop yield per hectare [71,72]. Statistically, the palm oil industry represents a world population of palm oil estimated to reach up to 63 metric tonnes of CPO annually, which is 36% of the total world production of vegetable oil [73]. As the world’s second largest producer, the Malaysian Government has introduced a campaign called “Love My Palm Oil” in order to disseminate information and to encourage Malaysians especially regarding this oil palm industry. However, recent finding on the presence of 3-MCPD in refined palm oil has triggered immense safety and health concern. The transformation of 3-MCPD might be related to elevated temperature and acid-activated bleaching during deodorization stage of oil refining [74]. Although there is no concrete evidence and toxicological data available on the severity of 3-MCPD on human, 3-MCPD is still deemed to be potentially harmful to human, as it is tested and proven to be on rats.

In 2017, the European Parliament announced a move to phase out and ultimately ban palm oil from entering their markets. The decision instigated a heated argument among palm oil stakeholders. One of the reasons behind the move is due to the presence of 3-MCPD in palm oil. Recent finding shows that the 3-MCPD, which is a process contaminant or a by-product formed during refinery processes, has the highest amount in palm oil compared to other types of vegetable oils such as olive, corn, sunflower and rapeseed [75]. Although the 3-MCPD is only perceived to be carcinogenic to humans without solid evidence, European Union has set a limit level of 2 µg 3-MCPD per kg bodyweight for HVP.

The exact mechanisms of 3-MCPD formation are still being carefully studied. It is suspected that the 3-MCPD is formed as a result of a combination of several different factors and conditions such as the magnitude of chlorination, acylation and the isomeric ratio between chlorinated compounds. Therefore, in order to mitigate, reduce and remove 3-MCPD from the vegetable oils, in depth understanding of the connection between possible contributing factors must be established. The establishment should include type and range of the possible precursors, as well as the influence of the processing conditions that lead to the 3-MCPD formation beyond tolerable limits [76].

## 3. Trigger Factor in 3-MCPD Formation

### 3.1. Precursors

The 3-MCPD level in palm oil is reported to be the highest among other vegetable oils, with numerous investigation have been conducted to determine the root cause of the formation of the contaminant, especially in palm oil [77]. It was noted that the 3-MCPD ester level is significantly higher in refined vegetable oils than in their unrefined counterparts, indicating that the trigger source of 3-MCPD formation might be due to the oil refining process [9,57,60,62]. In addition, the bound form of 3-MCPD level in the vegetable oil increased upon heating at 260 °C [61]. Based on the aforementioned literature, it can be presumed that a high temperature of above 200 °C during refining process is one of the culprits for 3-MCPD and 3-MCPD ester generation.

Nonetheless, a few reported that most 3-MCPD esters are developed during the oil deodorization [48,78]. A few other researchers reported that a large removal of MAGs, DAGs, phospholipids and glycolipids occur during oil degumming [63,79]. Therefore, another suspected precursor for 3-MCPD generation in palm oil is TAGs, which preferentially reacted with chlorine donor to form 3-MCPD esters. The TAGs undergo cyclic acyloxonium pathway, where an acyloxonium ion intermediate is formed [80]. Due to the inconclusive conclusion thus far on the 3-MCPD formation pathway, it might also be safe to say that 3-MCPD formation is more of a multivariate issue than just a straightforward topic of discussion.

#### 3.1.1. Chlorine

Chlorine-containing compounds exist in the form of either inorganic or organic. Inorganic chlorine salts of calcium chloride (CaCl_2_), magnesium chloride (MgCl_2_), iron (III) chloride (FeCl_2_) and iron (III) chloride (FeCl_3_) originate from fertilizers and irrigation process [80]. FeCl_2_ and FeCl_3_ were reported to have higher contents compared to other sources of inorganic chlorine. The occurrence of organochlorines in CPO prior to processing indicates that the chlorinated compounds might be present in the oil palm fruitlets even before harvesting [81]. The correlations between total chlorine content in vegetable oil and 3-MCPD level have also been established by many researchers [80,81,82,83,84,85]. It was suggested that organochlorines might indirectly act as a chlorine donor during oil deodorization process, which takes place at a temperature above 180 °C. During the process, the sum of organochlorines depleted as the sum of MCPD diesters increased and HCl was formed. Therefore, HCl is suspected to be one of the reactive compounds contributing to the formation of 3-MCPD [63,79,80,81]. It has also been identified that the 3-MCPD ester level increases with the addition of ionic bound chlorine, for instance tetrabutylammonium chlorine (TBAC) during the modelling of deodorization for vegetable oil [82]. A similar finding, involving a laboratory scale of CPO physical refining highlighted the potential of natural organochlorine as a chlorine precursor due to its oil solubility, which inhibits its removal during rinsing step with water [85].

A previous study reported on the effect of adding oil-insoluble sodium chloride (NaCl) and oil-soluble tetra-n-butylammoniumchloride (TBAC) followed by heating under standardized conditions of 240 °C for 2 h in order to test for the effect of soluble and insoluble chloride towards formation of 3-MCPD [83]. The results showed that both additions of soluble and insoluble source of chloride assisted a surge in the total contents of 3-MCPD esters and its related compounds. This result indicates that the appearance of organic and inorganic chloride-containing compounds contributes to the overall formation of 3-MCPD esters and mitigation of the compounds has to be performed in order to eliminate the source of 3-MCPD precursors.

The highest amount of 3-MCPD was reported to form in the presence of NaCl as the source of chlorine released by 1-monopalmitin in soybean oil [86]. Most of the inorganic chlorine precursors were identified in the oil palm plantation, where the first source of those precursors is the fertilizers. Conventionally, among fertilizers used in the oil palm plantation are ammonium chloride, NH_4_Cl and potassium chloride, KCl [87]. In addition, the herbicides used in the plantation, namely diuron, 2,4-D amine, dicamba and fluroxypyr also contained chlorine compound [88,89]. As most of the herbicides used in the plantation are water soluble, the oil palm fruitlet is likely to be exposed to the chlorine compound through nutrient uptake by the palm trees and through leaching process, where the chlorine compound dissolved in groundwater which will then be absorbed by the palm trees during cultivation [90].

The irrigation water used in the oil palm plantations is also a possible source of chlorine precursor [91], in addition to the treated wastewater from the treatment facilities that used FeCl_3_ as the flocculant [92]. Moreover, the bruising of fresh fruit bunches (FFBs) was identified to correspond with the increase in FFA content in the palm oil during harvesting and transportation to the mills [93]. The FFA formation is equivalent to the formation of DAGs and MAGs, which influence the formation of 3-MCPD. In addition, higher FFB bruising was reported when the bunches were transported by old trucks compared to the new ones, where the bruising index were 2.01 and 1.82, respectively [94]. This indicates that method of transportation indirectly affects the formation of 3-MCPD due to the increasing among of FFA when the bruising of FFBs worsen.

The increase in 3-MCPD ester formation was reported when NaCl was added into the glycerol when heating occurs [65]. NaCl is commonly tested in various researches as it is commonly used in food industry as food preservative and flavoring agent [95]. It was also reported that addition of NaCl in palm oil has caused the formation of 3-MCPD [83]. Since then, increasing variety of inorganic chloride including KCl, NaCl, FeCl_2_, FeCl_3,_ ZnCl_2_, CuCl_2,_ CaCl_2_, AlCl_3_ and NH_4_Cl were examined to identify their effects on 3-MCPD formation in vegetable oil. Various results were reported in this context according to the literatures discussed in this work [96,97,98,99,100]. While some studies found that all the chloride exhibited the effects on 3-MCPD formation, there was a consensus that the metal ions, particularly Fe^3+^ and Fe^2+^ enhanced the 3-MCPD formation through their catalytic effects on the reaction. FeCl_3_, for example would react with glycerol tristearate to form 3-MCPD esters, hence supporting the reactive mechanism of 3-MCPD formation directly from TAG in addition to the mechanism involving DAG [80,97,98].

**Figure 2:**
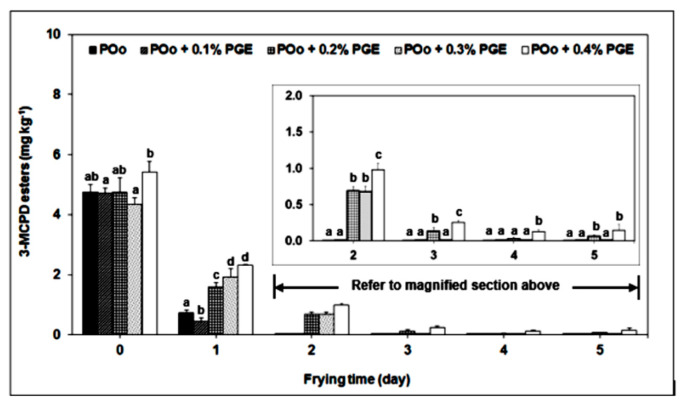
Degradation of 3-chloropropane-1,2-diol (3-MCPD) esters in palm olein (POo) added with polyglycerol fatty acid esters (PGE) during repeated frying. The 3-MCPD esters for frying times between day 2 and day 5 can be referred to in the magnified section to improve clarity. One-way analysis of variance (ANOVA) was used to indicate the significant difference between the control experiment (POo without PGE) and each frying interval (*p* < 0.05), as shown by different lowercase letters [102].

The decomposition of organochlorines in unrefined palm oil upon thermal treatment ignites interest on 3-MCPD esters formation through nucleophilic substitution between TAGs and HCl as the chlorine donor [80]. The chlorine-containing compounds transform throughout the agricultural process from palm oil growth, maturation and harvest [58]. Different combinations of factors, such as different sources of chloride donors, different processing conditions—for instance operating temperature—different isomeric ratios of 3-MCPD to 2-MCPD and other external factors may contribute to the inconsistent trends of 3-MCPD formation [101]. For instance, a study was conducted to evaluate the effect of adding anti-clouding agent in the form of polyglycerol fatty acid esters (PGE) on the formation of 3-MCPD esters during scheduled French fries frying time in palm oil (palm olein) at a temperature of 180 °C for five consecutive days. The change in chloride concentration was also periodically monitored. Results showed that the concentration of 3-MCPD esters in palm olein decreased from day 1 to day 5 during recycled frying as shown in Figure 2, while the concentration of chloride remains significantly unchanged throughout the experience. Palm olein containing PGE content higher than 0.1% showed slower decay rate of 3-MCPD esters suggesting that the addition of PGE decelerated the decay rate of 3-MCPD esters. PGE, in particular exhibits a wider range of polarity with 6 to 11 range values of hydrophilic-lipophilic balance (HLB), which shows higher tendency of being hydrophilic than hydrophobic. It is suspected that the higher polar compounds in PGE in the form of oxidized and polymerized TAGs led to the formation of an intermediate acyloxonium ions, which subsequently led to the formation of new 3-MCPD esters. An anti-clouding agent helps in improving the clearness and visual quality of the oil [102].

On the other hand, another study was conducted using frying sample of chicken meat at temperatures of 160 and 180 °C, with the addition of different concentration of NaCl showed otherwise. The results showed a consistent increment of 3-MCPD esters formation with the elevation of frying temperature and concentration of NaCl from 1% to 3% [103]. This shows the inconclusive trends of chloride-containing compounds as precursors to the materialization of 3-MCPD and its esterified form. The 3-MCPD formation is suspected to depend on various physicochemical properties and the interaction between components in the system.

#### 3.1.2. Acylglycerols

Acylglycerol, also known as a glyceride is an esterified form of glycerol. Glycerol has three hydroxyl functional group (-OH), and classification of acylglycerol depends on the number of fatty acids replacing the hydroxyl group. In general, there are three different lipid groups of acylglycerols in vegetable oils, namely MAGs, DAGs and TAGs. In palm oil, for example, TAGs account for about 88 to 96% of the main lipids [80] whereas DAGs account for about 4 to 12% [104]. MAGs content is quite low compared to DAGs and TAGs, and easily volatilized during high temperature process. Therefore, MAGs presence might not be as impactful as the other two groups of acylglycerols when exposes to chloride-donor heated at a temperature of above 180 °C [105]. The groups of acylglycerols are able to form 3-MCPD esters under oil deodorizing conditions, thus making them the 3-MCPD precursors. 3-MCPD esters formed faster than DAGs, and the removal of DAGs from the vegetable oil corresponds to significant reduction of 3-MCPD during the refining process [82]. In addition, TAGs preferentially react with the chlorine donor to form 3-MCPD ester compared to the other acylglycerols, which are potential precursors to 3-MCPD mono and diesters formation through cyclic acyloxonium intermediate under the conditions of low moisture and high temperature [80,98].

Comparing palm oil with olive oil and avocado oil, which fall under the same category of fruit oils for their capability of forming 3-MCPD, it has been observed that olive oil and avocado oil show lower probability than palm oil. One of the possible reasons is due to the content of DAGs in palm oil is much higher than in olive oil and avocado oil [53].

### 3.2. Refining Process

The oil refining process is crucial for ensuring the quality of oil is safe for consumption. The effects of degumming and bleaching processes towards the 3-MCPD formation in the vegetable oil indicates that the increasing amount of phosphoric acid (H_3_PO_4_) used in the acid degumming step can result in an increment of 3-MCPD formation [78]. Therefore, in order to minimize and mitigate the 3-MCPD formation in palm oil during degumming process, it is suggested to opt for water degumming process, which can also help to remove chloride-containing compound from the oil system [66].

The correlation between the acidity of the bleaching earth used in the refined palm oil shows that the acid-activated bleaching had reduced the pH of the palm oil, hence causing the elevation in the 3-MCPD content in the oil [78]. In contrast, having a higher average pore size and volume as well as rougher pore surface, acid-activated bleaching earth possesses superior adsorptive capability compared to the neutral bleaching; hence, it is able to remove the precursors in the palm oil [66,75].

A laboratory-scaled deodorization of rapeseed oil was conducted in the temperature range of 180 to 270 °C for 20 min and results showed a substantial increase of the 3-MCPD formation from 210 °C onwards [49]. Therefore, it is highly expected for 3-MCPD formation to occur during deodorization of palm oil with an operating temperature in the range of 240 to 270 °C, higher than the deodorization condition for rapeseed oil. However, an initial study conducted to investigate the effect of varying time (1, 3 and 5 h) and temperature (180 and 230 °C) during palm oil deodorization reported no significant increase in 3-MCPD esters. In fact, the 3-MCPD esters showed a slight decrement after tested in all possible deodorization time and temperature settings. Glycidyl fatty acid ester (GE), on the other hands showed increment for temperature setting of 230 °C, increasing steadily for deodorization time from 1 to 5 h [63]. A possible explanation to the contradicting results obtained might indicate that the 3-MCPD esters formation correlates with GE decomposition, which is another processing contaminant dependent to the deodorization temperature [49].

GE is another main contaminant in refined vegetable oils, mainly formed during the deodorization step in the refining process. GE is also considered as potentially carcinogenic, with a similar threat imposed by 3-MCPD. Bidirectional conversion between 3-MCPD and glycidol, as well as the esterified forms of 3-MCPD esters and GEs also occurs during the refining process, where 3-MCPD is partially converted to glycidol during the transesterification step and glycidol is partly converted to 3-MCPD during the derivatization step. Under the acidic condition, the transformation rate of glycidol to 3-MCPD is higher than the conversion of 3-MCPD into glycidol in the presence of chloride-containing compounds [58,62,80,84,106].

The scope of study on the deodorization process was further expanded, where the effect of different retention time during deodorization process on 3-MCPD formation was also considered. However, the effects of different temperature and retention time were concluded to be rather complex. It was reported that the retention time factor was critical at a deodorization temperature of 250 °C and further intensified as the temperature increases up to 270 °C [77]. It was also reported that the 3-MCPD formation during deodorization in the palm oil decreased with time after undergoing deodorization for 24 h. This was due to the decomposition of 3-MCPD ester through dechlorination. However, elevated thermal process in palm oil processing was not possible as it will affect the oil quality through hydrolysis and oxidation [58].

### 3.3. Other Factors

In addition to the precursors discussed in the previous section, there are additional factors or conditions that act as driving force to the formation of 3-MCPD. These factors are related to the oil quality itself, as well as the processing conditions. The root cause analysis of several different factors in the formation of MCPD esters covers from the upstream processing of soil conditions, fertilization and irrigation, harvesting and downstream processing the refined palm oil [52].

In reference to the root cause analysis, soil condition in the plantation is one of the factors contributing to the 3-MCPD formation. Saline soils, which can be found near the sea or river mouth that is constantly swamped by sea or brackish water, contains high level of chloride salts [58,104]. It was also reported that FeCl_3_ acts as the convention coagulant in soil irrigation step to achieve suitable soil moisture for oil palm tree planting. This might result in absorption of the chloride-containing compound in the fruitlet due to soil condition, and therefore explains the presence of FeCl_3_ and FeCl_2_ among inorganic chlorides in the CPO [81].

It is a common practice to utilize fertilizers for plant growth. Potassium chloride (KCl) is one of the widely used fertilizers in oil palm planting [107]. It is also one of the cheapest potassium fertilizers. The sustainable practice of using oil palm waste as fertilizer in the plantation could potentially enrich the chloride ions within the plantation [108].

The possibility of producing palm oils with a reduced potential of forming 3-MCPD has been reported when performing under careful harvesting method. For example, companies in Malaysia have reported a significant reduction in its capability of forming 3-MCPD esters and related compounds. The goal was achieved by optimizing route and shortening the transportation distance between harvest and CPO production [58]. By reducing the time interval between harvest and CPO production, the overall quality of the FFBs can be preserved and therefore the free fatty acids and acylglycerols contents, which are precursors to the 3-MCPD formation can also be minimized [109].

The use of acidic degumming and acidic bleaching corresponded with a higher 3-MCPD ester content in the bleached palm oil (BPO) compared with water degumming and neutral bleaching [78]. The modified commercial palm oil extraction process by utilizing an evaporator for water-oil separation also yielded an acidic crude oil, with pH 4, and the in-vitro thermal treatment of the obtained oil resulted in a high 3-MCPD ester level [110].

FFA content is one of the important components in vegetable oils [111]. In the case of palm oil, the FFA content is highly dependent on the harvesting time and method, and the storage period of the fruit bunches. The optimization of these factors can result in a lower potential of 3-MCPD formation under meticulous harvesting methods [109]. In addition, harvesting over ripe fruit bunches resulted in elevated FFA amount in the extracted oil. The bruising on the palm fruits during harvesting helped to initiate the reaction between the oil released from the vacuole with the hydrolyzing enzyme, thus producing FFA. As FFA could possibly acts as positive hydrogen ion (H^+^) donor contributing to the formation of 3-MCPD ester, the FFA reduction test showed a reduction of 3-MCPD ester formation [112].

Numerous studies have also been reported on the significant effects caused by the oil refining conditions toward 3-MCPD esters formation. It has been acknowledged that 3-MCPD ester can potentially be formed upon thermal treatment of oil samples at an elevated temperature of more than 180 °C [81]. As temperature setting has been concluded as one of the significant factors in the 3-MCPD ester formation, many researchers have conducted extensive studies on the formation of 3-MCPD at the vegetable oil refining process, specifically on the targeted process involving high temperature. Many researchers have reported the formation of 3-MCPD ester in the vegetable oil during deodorization step [48,63,78,82,85], which operates at temperature between 220 and 260 °C [113]. Interestingly, a study on the thermal stability of 3-MCPD esters during deodorization process reported the degradation of the esters after 24 h of thermal treatment due to dechlorination process of the esters to form glycerides [57].

Furthermore, there are studies showing that the degumming and bleaching processes during palm oil refining also showed significant effects on the formation of 3-MCPD ester [78]. Similar results have also been obtained, where water degumming was reported to be able to remove chlorinated compounds from the oil and thereby helped in reducing the precursors content for 3-MCPD esters formation [66]. The amount of phosphorus acid used in the degumming process as well as the acidity of the bleaching earth have also been reported on its ability to affect the oil acidity, which explains the correlation between acidity of the refined oil and the 3-MCPD ester formation during refining process [50,78].

## 4. Measurement

Several analytical methods of quantification to detect and quantify the concentration of 3-MCPD in the oil sample have been established, which involve either a direct method or an indirect method. Most methods used indirect method, which involves separation of 3-MCPD from its esterified form via transesterification either in acidic condition [114] or in alkaline condition [115,116,117]. After releasing 3-MCPD from its bound form, the free 3-MCPD is purified, derivatized and quantified using gas chromatography-mass spectrometry (GC-MS) [117]. The direct method, on the other hand, involves direct determination of 3-MCPD in its free, unbounded form. In other words, the direct method involves determination of a single fatty acid ester and quantification of 3-MCPD is measured using liquid chromatography-mass spectrometry/mass spectrometry (LC-MS/MS) method [118].

Another possible 3-MCPD detection method is an electrochemical method, which involves detection of the targeted analyte using electrochemical process. The principle behind the detection is inherently different than the chromatographic method. The electrochemical method involves applying specified voltage or current over a capillary or medium filled with electrolyte that can assist electrochemical reaction via electroosmotic flow (EOF), which allows an electrophoretic separation for the detection of the targeted analyte, for instance 3-MCPD [119]. Examples of electrochemical techniques include cyclic voltammetry (CV), linear sweep voltammetry (LSV), and differential pulse voltammetry (DPV).

### 4.1. Analytical Measurement

Several analytical measurement methods have been established for the determination of 3-MCPD and its ester forms. The commonly used analytical methods can be further classified as direct and indirect methods, which subsequently require LC-MS and GC-MS, respectively to perform quantification of the 3-MCPD. The principles behind Association of Official Analytical Chemists (AOAC) Official Method 2000.01 to determine 3-MCPD content in foods and food ingredients involve adding an internal standard called 3-chloro-1,2-propanediol-d5 (3-MCPD-*d5*) deuterated isomer to the sample test solution for example palm oil sample, followed by addition of salt solution [120]. Next, the mixture is thoroughly mixed to achieve homogeneity, and sent for sonication. The contents of an Extrelut^TM^ refill pack subsequently added and mixed thoroughly, before transferring the mixture to a glass chromatographic tube, with the nonpolar components being eluted with a mixture of hexane and diethyl ether. The sample solution containing 3-MCPD contaminant is eluted with diethyl ether, and the extract is concentrated, derivatized and analyzed by GC-MS. The concentration of 3-MCPD is expressed in mg per kg [121].

Since detection of 3-MCPD is a critical step towards determining the quality of the vegetable oil, evaluation of methods used by laboratories has to be done. Therefore, a rigorous comparative study and investigation toward the capabilities of food control laboratories to quantify the 3-MCPD in vegetable oils was carried out by the Institute for Reference Materials and Measurements (IRMM) of the European Commission’s Joint Research Centre (JRC) [121]. The study was conducted using the same test materials of refined palm oil, extra virgin olive oil spiked with 3-chloropropane-1,2-dioleate and 3-MCPD standard solution in NaCl by laboratories from EU Member States, Switzerland and Macedonia using each of their own analysis methods. As a result, the participants’ satisfactory level for determining 3-MCPD content in palm oils was significantly lower in comparison with 3-MCPD determination in spiked extra virgin olive oils [121].

In order to quantify 3-MCPD esters in lipid environment, two (2) analytical chromatographic approaches may be applied; direct or indirect method. To monitor concentration of 3-MCPD esters in lipid samples, indirect methods are more frequently applied, based on either acidic or alkaline transesterification reaction in order to release free chloropropanediols from the esterified form, followed by purification (using liquid-liquid extraction), derivatization usually using phenylboronic acid (PBA), heptafluorobutyrylimidazole (HFBI) or using N,O-Bis(trimethylsilyl)trifluoroacetamide + trimethylchlorosilane and subsequently quantification by GC-MS [121,122]. However, this indirect approach requires multistep sample preparation, which can possibly trigger side reactions, but is still widely applied in quality control laboratories because it is reliable and relatively simple in regards to chromatographic separation.

#### 4.1.1. Direct Methods

The principle behind the direct methods involves direct determination of 3-MCPD content, which includes monoesters and diesters, followed by subsequent quantification of the 3-MCPD using liquid chromatography-mass spectrometry (LC-MS) method [123]. According to International Life Sciences Institute (ILSI), formation of 3-MCPD esters, which are formed at high temperature during refinery process of oils and fats, involves the transformation of a cyclic acyloxonium ion from triacylglycerol followed by reaction with chloride ions [118]. It is also reported that the main factor contributing to the formation of 3-MCPD esters are the presence of chloride ions, glycerol, tri-, di- and monoacylglycerols with the combination of temperature and time [124].

Direct determination of MCPD esters in vegetable oil can be achieved using liquid chromatography-time-of-flight mass spectrometry (LC-TOFMS) method. Comparing the result with an indirect method by German Society for Fat Science (DGF) showed that 3-MCPD content measured using DGF method consistently reports better sensitivity compared to LC-TOFMS method, which used sodium acetate as the mobile phase. The selection of sodium as a mobile phase also exhibited a disadvantageous impact on the MS system as it requires quick cleaning after each use [118].

A direct measurement of 3-MCPD in oils and fats without any derivatization treatment was presented using HPLC method with 1,2-dioleoyl-3-chloropropanediol and 1-stearoyl-3-chloropropanediol standards as reference standard substances. The linear range concentration was determined to be in between 0.01 to 0.28 mg per mL, whereas the limit of detection (LOD) and limit of quantification (LOQ) were 3.43 and 5.71 µg per mL for 1,2-dioleoyl-3-chloropropanediol and 2.55 and 5.66 µg per mL for 1-stearoyl-3-chloropropanediol, respectively [125].

Molecular imprinting is a promising strategy to enhance the selectivity of the sensor performance towards the targeted analyte and to decrease the effect of interfering elements inside the material matrix [126]. Most of the molecular imprinting method incorporate polymers, and the material is called a molecularly-imprinted polymer (MIP). MIP can be made to match the size and shape of the template molecules and to exhibit characteristic of an excellent detection of targeted element, which can improve the overall performance of a 3-MCPD detector or sensor [127,128,129,130].

#### 4.1.2. Indirect Methods

Determination of 3-MCPD content is mostly accomplished using this indirect method, which involves conversion of complex 3-MCPD esters into single 3-MCPD compound that can be detected analytically. The protocol of performing this indirect analytical method includes hydrolysis of esters or transesterification, followed by derivatization of free 3-MCPD, extraction of the derivative, and determination of 3-MCPD using GC-MS. It is difficult to distinguish 3-MCPD by detection of MS due to its low molecular weight. While the derivatization process is required due to the low volatility and high polarity of 3-MCPD. The process will prevent an undesired interaction, which can affect the peak formation and sensitivity of the targeted analyte [120]. Basically this method involves analysis of the sum of 3-MCPD esters together with its content in free form, and the result was expressed as total 3-MCPD [122]. Methods based on solid-phase extraction, derivatization followed by GC-MS analysis usually allows quantification of 3-MCPD at the µg per kg level. Despite modifications made at any stage during indirect method determination of 3-MCPD, basic protocols are still followed: addition of internal standard either in free or esterified form of 3-MCPD-*d5* deuterated isomer, transesterification, salting out using neutralizing compounds, derivatization of 3-MCPD and finally GC-MS determination [131]. Despite the reliability of the GC-MS method, it is a lab-based, expensive, time consuming and requires skilled personnel to perform the measurement. Therefore, there is a need for an in-situ, portable and facile method that can be done on site, and is able to produce fast and reliable results.

For hydrolysis of esters, the method can be performed under acidic condition, alkaline condition or under enzymatic action. The acidic-based method using concentrated sulfuric acid in methanol at 40 °C for a 16-h procedure resulted in high levels of 3-MCPD in bound form [120]. However, this method produced an insignificantly higher 3-MCPD value. The main factor is due to the addition of chloride ion from 1% NaCl that elevated the formation of 3-MCPD esters. Methoxide ion (CH3O-) is a strong base ion, which acts as a strong nucleophilic agent. In the 3-MCPD environment, the presence of methoxide ions can result in 3-MCPD decomposition by nucleophilic substitution [118]. Therefore, the modified method using transesterification with 0.5 M of sodium methoxide solution in anhydrous methanol shows a significant value in 3-MCPD contents. Therefore, in order to compensate the decomposition, addition of isotope-labeled 3-MCPD denoted as internal standard is essential [60]. This issue was resolved in DGF standard method by eliminating the GE removal step and using sodium bromide (NaBr) instead of NaCl during extraction process [132]. The method reported to have a recovery of the internal standard averaged around 63% and is subsequently standardized by German Society for Fat Science (DGF) as DGF Standard C-III 18 (09) [118].

Derivatization of free 3-MCPD is done using either PBA or HFBI followed by extraction with an organic solvent, for instance n-hexane. Next, quantification of 3-MCPD content is performed by isotope dilution GC-MS using a deuterium standard solution commonly labelled as 3-MCPD-d5. Usually the mass spectrometer is operated in either scan mode or selected ion monitoring (SIM) mode depending on the sample preparation [120].

## 5. Mitigation

**Figure 3:**
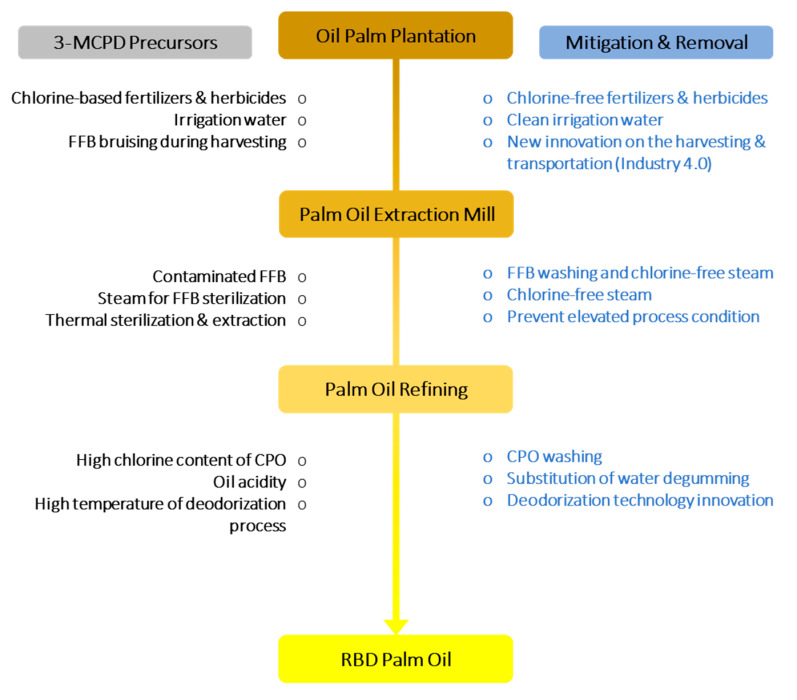
Proposed pathway for mitigation and removal of 3-MCPD from as early as in oil palm plantation management to the refining crude palm oil (CPO) process.

A guideline to control and mitigate this heat-induced compound of 3-MCPD formation in the palm oil production process has been established by focusing on the monitoring of 3-MCPD formation in the palm oil production process [52]. However, the analysis only emphasized on the scope within the oil plantation area. As discussed in earlier sections, 3-MCPD formation may occur before, during and after refinery. Therefore, it is also crucial to establish measures to control and reduce the occurrence, and the intensity of the 3-MCPD esters formation before it reaches consumers. Among strategies that have been reported in the literature include removal of raw material of critical reactants, and appropriate adjustment made to the refining process [53]. The control and monitoring of 3-MCPD formation in palm oil production process outlined in this review was summarized in Figure 3.

### 5.1. Plantation Management

The mitigation steps on 3-MCPD formation in palm oil should be executed from as early as in the plantation. The usage of chlorine-free fertilizers instead of conventional ones containing KCl and NH_4_Cl should be encouraged in the plantation. Changes in the plantation management practices are also needed so that the precursors contributing to the 3-MCPD formation can be eliminated and removed from the system. The conventional chlorine-based fertilizers should be replaced with chlorine-free fertilizers such as magnesium rich synthetic gypsum (MRSG), which gave a better oil quality without oil contamination and the plantation surrounding area [133]. The same principle should be applied to the herbicide as well.

Other ways to control and minimize the 3-MCPD occurrence in the oil palm plantations is to shorten and optimize the transportation distance between plantation area and milling site [53]. Industry experts suggested that the palm oil milling process for CPO production should be within 48 h to maintain minimum DAG (or FFA) content in the oil [134]. Furthermore, the industry should adopt innovations and mechanizations in oil palm harvesting and transportation in the plantation, which were reported to potentially enhance the overall quality of the fruit bunches upon arriving to the mills.

Such innovations included in-field evacuation of FFB using mini tractor installed with a high lift trailer that could load the FFB straight to the truck without leaving them at the roadside, thereby reducing the level of bruising [135]. The reduction of bruising could prevent the reaction between the oil with the inorganic chloride that was identified on the skin of the palm oil fruits [52]. The planters also needed to practice high maintenance of equipment and vehicles for FFB harvesting and transportation. This practice can prevent the exposure of palm oil to the metal ions such as Fe^3+^ that originated from the equipment and vehicles that could catalyze the reaction of 3-MCPD formation. Some significant changes to the current practices through the implementation of the fourth industrial revolution (Industry 4.0) may improve the plantation management [136].

### 5.2. Extraction and Refining Process Modification

One of the main precursors identified for 3-MCPD formation is chloride-containing compounds, either organochlorines naturally present in the soil or oil palm tree, or inorganic chlorine mainly from fertilizers. The discovery implies that mitigation of the chlorine sources must be performed prior to the sterilization step to remove the chlorine precursors on the skin surface of the fruits. One of the ways to remove this critical precursor is by washing off the FFB using water and ethanol [134]. Results show significant reduction of 3-MCPD content [53]. The effect of washing the raw material with water or ethanol (75%) prior to refining process is able to ease the formation tendency of 3-MCPD esters and related compounds formation by 20% and 25%, respectively. Therefore, this is one of the efficient ways of reducing and potentially eliminating the formation of 3-MCPD esters, as the possibility of the critical reactants to react with the raw materials has been eliminated.

The sterilization step in the palm oil mill is a process that involves heating and usage of water. Upon heating, the chlorine precursors can react with the TAG in palm oil via acyloxonium ion pathway or cyclic acyloxonium pathway [80]. The thermal processing during the CPO production can also contribute to the formation of FFA in the oil [137]. This not only translates to reducing oil quality but also accelerates 3-MCPD formation. A process improvement in FFB sterilization by installing a double roll crusher and a pre-heating unit has successfully reduced retention time and operating pressure of the sterilization process, hence is expected to reduce the risk of 3-MCPD formation according its mechanisms [138]. Moreover, a subsequence refining process by molecular distillation can also provide the purification of palmitic and oleic acid [111,139].

As various types of inorganic chloride were found to be present in the crude palm oil, mitigations at the extraction step would be expected to be highly effective [81]. Due to the solubility of the chloride compound in the washing medium, CPO washing using 1 to 5 *w*/*w*% water contributed to the reduction of 3-MCPD, and applied in the palm oil mills [135]. CPO washing at post-extraction stage was also reported to be effective in 3-MCPD formation reduction as the water-soluble precursors were removed [140,141]. This was further supported by a field study of utilizing washing process in one palm oil mill that showed an average chloride reduction of 84% in the CPO [134]. The suggested washing medium for this process included alkaline water and water/ethanol solution [79,134]. The palm oil producers should also avoid using chlorine-containing water in the sterilization process to prevent contribution to 3-MCPD formation.

As an alternative to the mechanical extraction, enzymatic processes also exhibited potential as a mitigation strategy in 3-MCPD reduction during palm oil extraction. The enzymatic extraction process operated under mild conditions with an extraction temperature of 50 °C as compared to mechanical extraction was reported to yield a comparable performance of reducing 3-MCPD formation, and subsequently produce CPO with a better quality [142,143].

In the context of chemical refining, the FFA content in the palm oil was primarily removed via neutralization process [140]. The neutralization of refined oil was conventionally done using calcium oxide, carbonate, bicarbonate and hydroxide compounds, which significantly exhibited a lower 3-MCPD content [63,77,82,83]. Moreover, since FFA has been identified as one of the precursors for the 3-MCPD formation, one of the ways to mitigate the FFA is through neutralization process prior to deodorization step using alkaline carbonates or hydrogen carbonates. This neutralization process is able to reduce the formation of 3-MCPD esters, with potassium salts showing better efficiency than sodium salts [82].

Several innovations in deodorization technology have been studied to identify their role in 3-MCPD reduction in refined palm oil. The dual deodorization process was reported to be able to obtain refined palm oil with lower 3-MCPD ester content with 65% and 75% 3-MCPD ester reduction under two different operating conditions. The dual deodorization process operates in a milder condition as compared to the conventional deodorization process, with the aim to prevent trans-fat formation and optimize vitamin E content in the oil for further extraction [53]. The optimization of the deodorization process, which was conducted using response surface methodology (RSM), showed 87.2% reduction of 3-MCPD content in refined palm oil, reducing 3-MCPD content from 2.9 mg per kg to 0.4 mg per kg [75].

Various studies involving the reduction of 3-MCPD formation in palm oil refining process have been conducted. For example, a 64% reduction of 3-MCPD ester content was achieved with water degumming process using 2 *w*/*w*% deionized water at 80 to 85 °C [78]. Reduction in 3-MCPD formation in palm oil by using water degumming is even better over acid degumming process. The mechanism that explained this removal was due to the polarity of the chloride compounds towards water. In addition, the usage of water over phosphoric/citric acid in the degumming process has helped to reduce the pH of the palm oil, thereby eliminate another factor that can induce the formation of 3-MCPD [66,77,140].

### 5.3. Post Refining Process

Another attractive measures in mitigating the 3-MCPD in palm oil involves the post-refining processes. A feasible approach was reported to remove 3-MCPD in its esterified form using adsorbent material [142]. Different types of adsorption materials have been used to remove polar components in frying oils [144,145,146,147,148]. The charge difference between the 3-MCPD esters and the TAGs in the oil allows the separation process to occur, with the help of the absorbents, for instance calcinated zeolite, and therefore successfully reduces the level of 3-MCPD esters in the palm oil [144]. Results also indicate that only two of the adsorbent materials, specifically the calcinated zeolite and the silicon calcium silicate, showed significant reduction of up to 40% compared to the initial concentration of 3-MCPD and its esterified form. In addition, the usage of additives such as rosemary extract and lipophilic tea polyphenol during deodorization was also able to achieve 82% and 75% reduction of 3-MCPD ester in the palm oil, respectively [148].

Additionally, an environmentally-friendly enzymatic approach to mitigate 3-MCPD formation was also introduced. The enzymatic approach was designed to remove 3-MCPD from aqueous and biphasic systems via an enzymatic conversion of 3-MCPD esters to glycerol, which is allowable to appear in food, using a halohydrin dehalogenase from *Arthrobacter* sp. AD2 and an epoxide hydrolase produced from *Agrobacterium radiobacter* AD1 [149]. Another enzymatic approach utilizing biodegradation of 3-MCPD is using *Saccharomyces cerevisiae*, tested on two (2) racemic 3-MCPD concentrations of 7.3 µmol/L and 27 mmol/L. Conversions of 68% and 73% were reported for racemic (R,S) 3-MCPD of 27 mmol/L and 7.3 µmol/L, respectively, with varying reaction time from 48 h up to 72 h. This method is viable, and therefore is another attractive and green approach to remove 3-MCPD from a system. However, it takes at least 48 to 72 h to show significant 3-MCPD reduction [150]. Table 1 summarized effects of mitigation processes on the total 3-MCPD reduction.

Another promising approach to mitigate the 3-MCPD formation is by adding antioxidants that can help in obstructing the formation of radical intermediates. A selected group of antioxidants that includes butylated hydroxyanisole (BHA), tert-butylhydroquinone (TBHQ), butylated hydroxytoluene (BHT), oleoresin rosemary and sage extract was tested in RBD palm olein undergoing deep frying of potato chips at 180 °C for 100 min in separate systems for three consecutive days. Results showed promising usage of antioxidants in mitigating the 3-MCPD formation with TBHQ and oleoresin rosemary having better efficiencies of inhibiting the 3-MCPD formation compared to the other antioxidants tested [151]. With dihydroxyphenols, TBHQ possesses higher antioxidant characteristics, having more hydrogen capable of delaying the radical intermediate formation prior to 3-MCPD esters formation, compared to BHA and BHT with monohydroxyphenols [151,152]. A further test has also been performed to analyze the effect of using an antioxidant combination of rosemary and tocopherol to the palm olein and soft stearin in reducing the 3-MCPD content. The results indicated lower 3-MCPD contents as depicted in Table 2. The concentrations of 3-MCPD esters prior to a baking experiment at a temperature of 160 °C for 20 min were between 3.15 to 4.19 mg per kg. The reduction of 2-MCPD and GE for palm olein and soft stearin by rosemary extract showed a significant relation to the formation of 3-MCPD [153].

The addition of antioxidants is indeed capable of mitigating the formation of free radicals formed during thermal treatment of palm oil-derived products by defying the formation of free radicals. These free radicals are able to react with 3-MCPD precursors to form 3-MCPD, a food contaminant that still haunts the lives of millions of refined vegetable oil producers and users, especially in palm oil industry.

## 6. Conclusions

Higher concentrations of 3-MCPD have been reported for refined vegetable oils and fats, particularly in refined oils compared to unprocessed oils, which supports the hypothesis that processing conditions play important roles in the occurrence and formation of 3-MCPD and its esterified bound form. Thermal treatment, presence of precursors, and other defining factors such as soil condition and harvesting procedures have been reported to be the possible trigger factors for the formation of 3-MCPD content, even beyond TDI. Therefore, it is important to continue researching for all possible pathway formations, mitigation and removal methods to ease 3-MCPD management, as it is still not fully understood to date. This is also to ensure that the processed and refined foods consumed by humans are safe and of the best quality. Improvements could also be done by upgrading or further fine-tuning the refining process technology, which can hopefully help to permanently reduce and eliminate the occurrence of 3-MCPD. More in-depth studies should also be conducted, to mitigate the formation of 3-MCPD and subsequently permanently reduce and remove the 3-MCPD, without tarnishing the premium quality of the vegetable oils, particularly palm oils.

## Figures and Tables

**Table 1:** Effects of mitigation on total 3-MCPD reduction.

Samples	Method	3-MCPD Concentration/Removal		Ref.
		Initial	Final	
3-MCPD and related substances	Adsorption technique using calcinated zeolite (10%)	6.59 ± 0.19 ppm	4.00 ppm	[144]
	Adsorption technique using synthetic magnesium silicate (10%)	6.59 ± 0.19 ppm	4.50 ppm	[144]
3-MCPD esters	Addition of rosemary extract	100%	82% reduction	[148]
	Addition of lipophilic tea polyphenol	100%	75% reduction	[148]
3-MCPD	Enzymatic removal with both *Arthrobacter* sp. AD2 and *Agrobacterium radiobacter* AD1 for 7.5 h	10.00 mM	2.9 mM	[149]
	Enzymatic removal with sequence of *Arthrobacter* sp. AD2 and *Agrobacterium radiobacter* AD1) for 3 h	10.00 mM	0.00 mM	[149]
Racemic (R,S) of 3-MCPD	Enzymatic removal with *Saccharomyces cerevisiae* at different initial concentration of 3-MCPD	7.3 µmol/L	68% conversion	[150]
		27 mmol/L	73% conversion	[150]

**Table 2:** Effects of different antioxidants (200 ppm) on the content of 2-monochloropropandiol (MCPD), 3-MCPD and glycidyl ester (GE) on the fats portion extracted from cake baked at 160 °C for 20 min with different shortenings [153].

Samples	Antioxidant	3-MCPD, mg/kg	2-MCPD, mg/kg	GE, mg/kg
	Butylated hydroxyanisole	3.439 ± 0.029 ^a^	2.334 ± 0.021 ^a^	1.987 ± 0.039 ^a^
Palm Olein	Rosemary	3.431 ± 0.065 ^a^	2.051 ± 0.010 ^b^	1.979 ± 0.029 ^a^
	Tocopherol	3.527 ± 0.189 ^a^	2.143 ± 0.105 ^b^	1.985 ± 0.047 ^a^
	Butylated hydroxyanisole	2.222 ± 0.028 ^a^	1.534 ± 0.114 ^a^	0.665 ± 0.001 ^a^
Soft Stearin	Rosemary	2.176 ± 0.032 ^a^	1.489 ± 0.040 ^a^	0.607 ± 0.032 ^b^
	Tocopherol	2.172 ± 0.023 ^a^	1.435 ± 0.012 ^a^	0.668 ± 0.010 ^a^

Values are the mean of two determinations from two replicate experiments ± standard deviation (*n* = 4). Mean values with different superscript letters in the same column are significantly different at *p* < 0.05.
